# Hurricanes and healthcare: a case report on the influences of Hurricane Maria and managed Medicare in treating a Puerto Rican resident

**DOI:** 10.1186/s12913-019-4630-z

**Published:** 2019-11-08

**Authors:** George Mellgard, David Abramson, Charles Okamura, Himali Weerahandi

**Affiliations:** 1 Department of Medicine, New York University Langone, New York, NY USA; 2 College of Global Public Health, New York University, New York, NY USA; 3 Department of Population Health, NYU School of Medicine, New York, NY USA

**Keywords:** Case report, Hurricane Maria, Puerto Rico, Managed Medicare, End stage renal disease, Special enrollment period

## Abstract

**Background:**

While Medicare is a federal health insurance program, managed Medicare limits access to healthcare services to networks within states or territories. However, if a natural disaster requires evacuation, displaced patients are at risk of losing coverage for their benefits. Previous literature has discussed the quality of managed Medicare plans within Puerto Rico but has not addressed the adequacy of this coverage if residents are displaced to the continental United States. We explore Hurricane Maria’s impact on a resident of Puerto Rico with chronic health problems, and the challenges he faces seeking healthcare in New York.

**Case presentation:**

A 59-year-old male with a history of diabetes mellitus type II, coronary artery disease, peripheral vascular disease status post right foot amputation, and end-stage kidney disease on hemodialysis was admitted in October of 2017 for chest pain and swelling of legs for 5 days. The patient had missed his last three dialysis sessions after Hurricane Maria forced him to leave Puerto Rico. In examining this patient’s treatment, we observe the effect of Hurricane Maria on the medical management of Puerto Rican residents and identify challenges managed Medicare may pose to patients who cross state or territory lines.

**Conclusions:**

We employ this patient’s narrative to frame a larger discussion of Puerto Rican managed Medicare and provide additional recommendations for healthcare providers. Moreover, we consider this case in the context of disaster-related continuity of care for patients with complex medical conditions or treatment regimens. To address the gaps in the care of these patients, this article proposes (1) developing system-based approaches for screening displaced patients, (2) increasing the awareness of Special Enrollment Periods related to Medicare among healthcare providers, and (3) creating policy solutions to assure access to care for patients with complex medical conditions.

## Background

On September 20th, 2017, Maria made landfall in Puerto Rico as a Category 4 hurricane [1]. By the next day, 0% of Puerto Rican residents had power and only 44% had access to potable water [2]. Hurricane damage further exacerbated existing healthcare disparities in Puerto Rico, particularly for those with End Stage Renal Disease (ESRD).

Disparities alone provide ample motivation for Puerto Ricans to seek health care in the mainland United States. Previous research has revealed a consistent lack of funding and access to timely.

care for Medicare and Medicaid beneficiaries living in Puerto Rico [3–5]. Stateside Puerto Ricans are more likely to receive preventive care and have higher utilization of health care services compared to Puerto Rican residents [6]. These disparities extend to the management of chronic kidney disease. Among individuals with diabetes and enrolled in managed Medicare, Hispanics in the mainland United Sates are more likely than Puerto Rican residents to undergo preventative measures including nephropathy screening [6].

Hurricane Maria created additional challenges for Puerto Rican patients with ESRD. Sources of water and power are required to power hemodialysis machines, create dialysate, and otherwise provide care for the 5332 Puerto Ricans using hemodialysis centers [7]. Yet by October 3rd, only nine of the 68 hospitals in Puerto Rico had been restored to the power grid [2]. With close to 700,000 Puerto Ricans already living in New York City as of 2016 [8], natural disasters such as Hurricane Maria have prompted further movement to the United States Mainland. Between October 3rd and 25th, 73,000 Puerto Ricans arrived in Florida to seek refugee from Hurricane Maria [9].

Inadequate healthcare, hurricane damage, and resultant displacement create new challenges in caring for patients with chronic health conditions. These issues are compounded by the high enrollment of Puerto Rican Medicare Beneficiaries in managed Medicare [10]. We now report a case in which Hurricane Maria forced a dialysis dependent patient to evacuate Puerto Rico in order to seek treatment. Additionally, we discuss possible shortfalls in the ability of out-of-state managed Medicare to cover necessary services for patients displaced by natural disasters.

## Case presentation

A 59-year-old male with a history of diabetes mellitus type II, coronary artery disease, peripheral vascular disease status post right foot amputation, and ESRD on hemodialysis was admitted to our hospital in October of 2017 for chest pain and swelling of legs for 5 days. Notably, the patient is a resident of Puerto Rico, but due to delays caused by Hurricane Maria, was unable to receive his last three dialysis sessions. As a result, 3 days prior to admission, the patient travelled to the continental United States to obtain dialysis.

In the emergency department, the patient had a temperature of 36.8 °C, blood pressure of 180/83, heart rate of 84, respiratory rate of 20, oxygen saturation of 96%, and was found to have lower extremity edema and right stump ulceration with distal necrotic tissue. Labs revealed a potassium level of 5.1, blood urea nitrogen of 67, anion gap of 9, creatinine of 9.990 and a Troponin T of 0.21. An electrocardiogram did not show peaked T-waves. The patient was admitted to reinstate dialysis and rule out acute coronary syndrome. Chest pain resolved after dialysis, and troponins trended down. It was concluded that the mild troponin elevation was likely demand ischemia secondary to fluid overload. To manage the right foot ulcer noted on admission, podiatry was consulted and recommended wound care services for the patient. Social work also recommended outpatient transportation to dialysis given his limited mobility. The patient was then medically cleared for discharge 2 days after admission, after five inpatient dialysis sessions.

Given the recommendation for these outpatient services, social work was consulted for discharge planning. Though the patient was insured through Puerto Rican managed Medicare, the patient’s coverage was not transferable for these services in the continental United States. The team consulted a hospital financial officer who found that to be eligible for New York State Medicaid, the patient would need to change his social security information, mainly his address, to reflect New York residence. While New York State Medicaid remained a potential option, the application process can take as long as 2 months and requires that the applicant maintain state residence for at least a month. This process was therefore not feasible for our patient who wanted to eventually return to Puerto Rico after being discharged to his parents’ home in Brooklyn. As a result, 9 days after admission, the patient was discharged to his parents’ home with local outpatient dialysis set up, but without access to either outpatient wound care or dialysis transportation. Ultimately, the patient was readmitted back to our hospital a month and a half later for non-ST elevation myocardial infarction.

## Discussion

Given the frequency of natural disasters prompting evacuations and displacement from communities across different U.S. states and territories, it is important to study the ability of managed Medicare to adequately cover health care services across these lines. Previous studies have suggested that managed Medicare plans in Puerto Rico have lower quality of care but none have explored their ability to provide adequate coverage for patients arriving in the mainland United States after a natural disaster [4, 6]. This report hopes to provide a novel perspective on Hurricane Maria’s influence on a Puerto Rican evacuee with managed Medicare and the challenges he faces seeking healthcare in New York. This patient’s case suggests a larger story as well: the difficulty in assuring continuity of care in a disaster’s aftermath for patients with complex medical conditions or medical regimens, even ones with federally-sponsored insurance.

Healthcare consequences related to natural disasters are not uncommon in the literature. Hurricanes such as Hurricanes Katrina and Sandy have a history of disrupting health care infrastructure [11–15]. Such disruptions can increase the number of diagnosed medical conditions in patients; and specifically for ESRD patients, lead to a higher likelihood of dialysis patients missing their sessions and being hospitalized [11]. Our case highlights not only the consequences of a hurricane but also how the inflexibility of managed Medicare may further exacerbate healthcare challenges for this vulnerable group.

Managed Medicare is a staple of Puerto Rican health insurance. Puerto Rican enrollees in Medicare elect managed Medicare at a rate 42.1% higher than U. S enrollees nationally [10]. Managed Medicare appeals to low-income Puerto Ricans because it subsidizes the premium for Medicare Part B and offers lower cost-sharing options [6]. However, there is little in the literature discussing how managed Medicare’s inability to cover services outside a resident’s state or territory may affect the delivery of necessary services in the wake of a natural disaster.

This case ultimately demonstrates how disconnects in the healthcare system can create additional challenges for displaced populations. Of note, our patient was previously receiving home wound care in Puerto Rico prior to Hurricane Maria. Had the patient not elected a managed Medicare plan, he would have received wound care services upon hospital discharge through Medicare Part B. It was only the combination of the hurricane and his insurance that denied him this essential service upon his evacuation to New York.

**Fig. 1:**
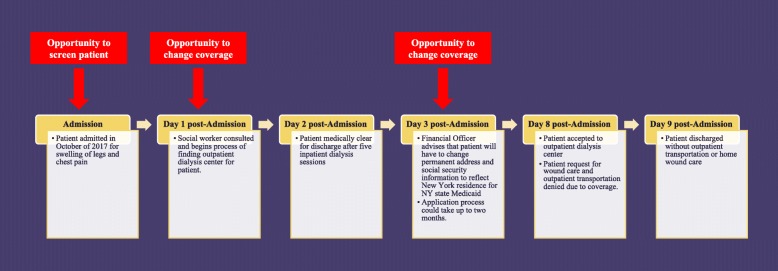
Description – Opportunities for Intervention

There are systems based approaches that could have addressed the coverage challenges faced by this patient (See Fig. 1). One opportunity could have been a point of control at hospital registration during which the hospital might identify him as a patient uprooted by Hurricane Maria. At this point, a social worker could be alerted to evaluate him and determine his eligibility for the Medicare Special Enrollment Period. Created by the Centers for Medicare and Medicaid in September of 2017, this Medicare Special Enrollment Period supports Americans affected by Hurricane Harvey, Irma, or Maria and allows them to switch managed Medicare plans outside annual enrollment times [16]. The patient and social worker were not aware of this possibility and the patient did not want to switch his coverage to a local managed Medicare plan. Alternatively, our patient could have been enrolled in New York Medicaid. However, enrollment in New York Medicaid requires patients to switch their permanent address to a New York address. Our patient planned to return to Puerto Rico within the next couple months. Consequently, this latter approach is limited if patients see their move as temporary and are unwilling to change their permanent address as required.

We suggest that managed Medicare provided unnecessary challenges for our patient and that further steps should be taken to ensure this vulnerable patient population always receives appropriate coverage of outpatient services. In this situation, the patient became responsible for recognizing that displacement qualified him for the Special Enrollment Period. Without direct intervention, however, the patient was never enrolled as neither he nor the hospital staff were aware that displacement due to a natural disaster qualified as a major life event to adjust Medicare coverage [17].

These challenges are not isolated to a single hospital admission. Post-Hurricane Maria, our hospital has seen a significant increase in hospital admissions of patients with insurance linked to Puerto Rican zip codes compared with the previous year. Continuity of care is not just important for patients on dialysis or for other patients requiring regular outpatient care such as intravenous infusions for cancer patients on chemotherapy, but for patients with highly prevalent chronic conditions that require regular follow up such as diabetes [18]. The risks facing these patients with chronic illnesses in the wake of hurricanes are well documented. In the days following Hurricanes Katrina and Rita, chronic illnesses accounted for 33% of hospital visits in New Orleans [14]. Moreover, Puerto Ricans with chronic illnesses remain vulnerable to disruptions of medical continuity secondary to disaster evacuations, even when they seek healthcare in the continental United States [19, 20]. Such delays in treatment like chemotherapeutics and post-surgical care can lead to preventable hospitalizations and increased morbidities beyond those documented in Puerto Rico.

The relative frequency of natural disasters in the United States and its territories suggests that the displacement of patients with chronic illnesses is not uncommon [21, 22]. Given the potential for patients to be uprooted during these times, these cases suggest hospitals take on a greater role in screening disaster patients and enrolling them in appropriate Medicare plans. We recommend that hospitals take a greater role in educating multidisciplinary healthcare teams on how displaced out-of-state managed Medicare patients can switch their insurance plans. We also propose limiting state restrictions on managed Medicare for patients affected by natural disasters. Such changes would allow patients forced from their homes to continue their enrollment in healthcare services without having to change plans. Ultimately these processes may ensure that these patients will receive seamless coverage for their care regardless of location.

Our approach has several potential weaknesses. First, we confined our report to the experience of one individual. While this approach allowed us to use a narrative to frame a larger problem, this case may not be generalizable. Second, when the patient moved back to Puerto Rico, he was lost to follow-up and, therefore, we were unable to continue to follow his healthcare longitudinally. Finally, our discussion of Managed Medicare is limited to the context of natural disasters and does not consider the potential benefits it might have under normal circumstances. Our discussion does not also consider the complex issues of those who may be displaced from areas outside the United States, such as the Bahamas after Hurricane Dorian.

## Conclusion

Hurricane damage was inevitable; the healthcare consequences were preventable. Despite the fact that the patient had federally-sponsored insurance with specified coverage for ESRD, the type of insurance coverage was the primary factor contributing to the denial of essential outpatient services for this patient in the continental United States. A systems-based approach such as screening patients to identify those who qualify for the Medicare Special Enrollment Period may mitigate concerns surrounding coverage of outpatient services. Hospital intervention and policy changes may ultimately be warranted to ensure that these patients get correct and timely coverage outside of areas directly affected by natural disasters.

## Data Availability

Data sharing is not applicable to this article as no datasets were generated or analyzed during the current study.

## Abbreviation

ESRD: End Stage Renal Disease
